# Generative AI emotion recognition from bodily gestures and vocal tone reveals modality-specific performance and positivity bias

**DOI:** 10.1038/s41598-026-58013-z

**Published:** 2026-09-03

**Authors:** David Piterman, Hadar Dery, Kfir Bar, Gunther Meinlschmidt, Alon Geller, Dorit Hadar Shoval, Zohar Elyoseph, Elad Refoua

**Affiliations:** 1 Faculty of Education, University of Haifa, Haifa, Israel; 2 Efi Arazi School of Computer Science, Reichman University, Herzliya, Israel; 3 Clinical Psychology and Psychotherapy, University of Trier, Trier, Germany; 4 Department of Digital and Blended Psychosomatics and Psychotherapy, Psychosomatic Medicine, University Hospital of Basel, Basel, Switzerland; 5 Ruppin Academic Center, Hadera, Israel; 6 The University of Kiryat Shmona in the Galilee, Kiryat Shmona, Israel; 7 Department of Psychology, Bar-Ilan University, Ramat Gan, Israel

**Keywords:** Generative Artificial Intelligence (GenAI), Emotion Recognition, Social Cognition, Nonverbal Communication, Algorithmic Bias, Gemini Models, Neuroscience, Psychology, Psychology

## Abstract

Advances in generative artificial intelligence (GenAI) have prompted interest in its application in sensitive fields, including mental health. Yet the validity of its nonverbal social-cognitive abilities remains unclear. This study addresses two issues that probe GenAI’s social intelligence: its capacity to interpret nonverbal channels (bodily gestures and vocal tone) with accuracy comparable to humans, and whether accuracy of emotion recognition is different by emotional valence. Emotion recognition accuracy of three Gemini GenAI models (Pro 1.5, Pro 2 (Experimental) and Flash 2) were evaluated using the EU-Emotion Stimulus Set of bodily gestures and vocal tone depicting basic emotions. Model accuracy was compared to validated human benchmarks. Advanced GenAI models achieved near-human accuracy in recognizing emotions from vocal tone. However, their performance was significantly lower than that of humans in interpreting bodily gestures. GenAI models recognized positive emotions more accurately than negative ones. This bias was significant across all models for bodily gestures and in the advanced models for vocal tone. GenAI models exhibit a non-humanlike social-cognitive profile, excelling with positive emotions but struggling with the negative content. This finding carries profound clinical and theoretical relevance, highlighting the risks of integrating GenAI into sensitive fields and underscoring the need for continued validation.

**Supplementary Information:**

The online version contains supplementary material available at https://doi.org/10.1038/s41598-026-58013-z.

## Introduction

The advent and widespread adoption of Generative Artificial Intelligence (GenAI) technologies, driven by the rapid evolution of Large Language Models (LLMs), have ignited substantial public interest, demonstrating remarkable capabilities across a vast array of applications, including programming, legal analysis, education, healthcare, and psychology^1–3^. Since the public release of ChatGPT, reports indicate that hundreds of millions of users are now leveraging AI^4^, with many seeking informal mental health support^5–7^. While these initial applications relied heavily on text-based LLMs, the current study expands this focus by evaluating advanced, natively multimodal GenAI systems. This burgeoning reliance on GenAI, particularly in sensitive domains, necessitates dedicated research into the capabilities of these models^8,9^, specifically concerning their proficiency in emotion recognition. Social cognition, encompassing the processes by which individuals perceive, interpret, and respond to social information, serves as a foundational component of adaptive social functioning^10^. A core component of social cognition is mentalization, the capacity to understand mental states in oneself and others^11^. Impairments in these social-cognitive abilities are associated with various forms of psychopathology^12^. Furthermore, many psychopathologies are characterized by deficits in mentalization^13,14^, and mentalization-based treatment (MBT) has been shown to promote mental well-being and reduce symptoms^15,16^. The improvement of these skills is considered a potential mechanism of therapeutic change, and is therefore a primary target for clinical interventions^17,18^. The ability of GenAI to accurately recognize emotions could significantly enhance therapeutic practices by providing scalable tools for mental health professionals to assess and monitor patients’ emotional states in real-time, potentially augmenting interventions like MBT in clinical settings^19^. Evaluating the extent to which GenAI can perform functions related to social cognition, especially in areas like emotion recognition which rely on mental state understanding, is therefore a topic of increasing relevance with direct implications for improving access to mental health care and personalizing therapeutic approaches^19,20^.

A growing body of research demonstrates the advanced social-cognitive capabilities of GenAI across a wide spectrum of tasks. In the textual domain, studies have shown that GenAI not only matches but even surpasses human performance on complex tasks of emotional awareness. For example, GenAI outperformed humans in the accurate identification and articulation of nuanced emotional states, as demonstrated on the Levels of Emotional Awareness Scale (LEAS)^21^. Furthermore, on the same task, the system demonstrated “performance plasticity”, indicating its ability to adapt its responses to different personality styles^22^. These capabilities extend to the static-visual domain; GenAI models have achieved human-level performance on the “Reading the Mind in the Eyes” emotion recognition task^23^, and recent studies report that current state-of-the-art models, such as ChatGPT-4o, have managed to exceed the human performance benchmark on the very same task^24,25^. Beyond emotion recognition, these models also exhibit capabilities in Theory of Mind (ToM) tasks^26,27^ and in interpreting social cues from general images^28–30^. Even more impressively, these abilities are not limited to static stimuli. Recently, it has been found to perform in levels similar to, and even exceeding, those of humans in complex and dynamic social inference tasks, such as the Movie for the Assessment of Social Cognition (MASC), which combines verbal, vocal, and behavioral cues^31^.

Despite this impressive progress, several limitations temper the interpretation of these findings and highlight gaps in our understanding. First, a substantial portion of the research relies on static and decontextualized stimuli (e.g., text, single images), which do not faithfully represent the dynamic complexity of real-world social interactions^23,31,32^. Second, in studies that use complex, multimodal stimuli (like the MASC), the integrative nature of the cues makes it difficult, if not impossible, to isolate the distinct contribution of each sensory channel. It is unclear whether the model’s success stems from decoding vocal tone, body movements, facial expressions, or verbal content, leaving its operational mechanism as a “black box”. Third, various studies indicate the existence of systematic performance biases in the models. These biases range from the misrepresentation of mental disorders^33^ to a consistent tendency for the under-detection of negative emotions and psychological distress^32,34^. Furthermore, the latter is now recognized as a critical barrier to safe clinical implementation, highlighting the significant consequences of such algorithmic flaws^19^. For instance, under-detection of negative emotions could undermine the effectiveness of GenAI-assisted mental health tools, potentially leading to missed opportunities for early intervention in therapeutic settings^35–37^. Finally, the use of a low number of trials in some studies, and a reliance on a forced-choice format that simplifies the task of emotion recognition may inflate performance scores. Collectively, these limitations underscore the need for research that can systematically and controllably evaluate the fine-grained GenAI`s capabilities in processing distinct, dynamic, nonverbal channels. The present study was designed to directly address this critical gap by focusing specifically on nonverbal cues as conveyed through two primary modalities: vocal tone and bodily gestures.

The ability to accurately interpret vocal tone and bodily gestures is critical for navigating daily social relationships, fostering trust, and is foundational to social cognition and adaptive functioning^10,38,39^. Consequently, deficits in this domain are a hallmark of numerous psychopathologies, making the study of these channels vital for clinical psychology. In therapeutic contexts, accurate recognition of these nonverbal cues by GenAI could facilitate the development of automated systems for monitoring patient emotional states^35,40,41^, enabling clinicians to tailor interventions more effectively^19,20^ and provide real-time feedback during therapy sessions^41^. Importantly, these two modalities, so crucial for human interaction, are hypothesized to present formidable challenges for GenAI. Vocal stimuli, when isolated, can be deconstructed into relatively distinct acoustic parameters for analysis (e.g., tone, prosody, speech rate)^42^, a task that constitutes a classic challenge in pattern recognition, a domain where GenAI demonstrates core capabilities. Its strength lies in its ability to apply computational models that simultaneously analyze both the ‘musical’ and harmonic characteristics of the voice at a given moment, and the evolving prosody and intonation throughout the sentence^43^. While interpreting vocal stimuli may constitute a well defined computational problem for GenAI, interpreting bodily gestures is considered a far greater challenge, as it requires a holistic understanding of complex, high-dimensional, and context-dependent dynamic movements and postures^44^, which may strain the capabilities of current GenAI architectures^45^. Given these potential differences in complexity, exploring how GenAI performance varies across these modalities is a critical aspect of understanding their current capabilities and limitations^46^, particularly for applications in mental health care where precise emotional assessment can inform diagnosis and treatment planning^35,37,41^.

In addition to the modality‑specific challenges outlined above, contemporary discourse on GenAI foregrounds two intertwined concerns: (a) entrenched algorithmic biases that originate in the data and optimization objectives of large models, and (b) a growing tendency toward *“sycophancy”* the production of responses that conform to what the system infers the user wishes to hear. Empirical work has documented systematic demographic, cultural, and affective distortions in model outputs, from unequal gender‑ and race‑related misclassification rates^47^ through stereotyped representations of mental disorders^33^ to the under‑detection of negative affect and psychological distress^32,34^. Instruction‑tuning for human preference, while improving fluency and safety, further encourages agreeable or overly confident replies, masking uncertainty and reinforcing existing biases^48,49^. These dual forces data‑driven bias and compliance‑driven sycophancy pose a particular threat in mental‑health contexts, where overlooking subtle cues of distress or pain carries clinical stakes. For example, a positivity bias in GenAI could lead to underestimating the severity of a patient’s depressive symptoms, potentially delaying critical interventions in telehealth or automated therapy platforms. Against this backdrop, the present study asks whether GenAI systems exhibit a functional positivity bias in emotion recognition, operationalized as systematically higher accuracy for positive relative to negative emotions, thereby providing a principled test of bias and sycophancy in a clinically salient, non‑verbal domain.

In summary, the research literature indicates several key gaps: the need for studies based on larger and more diverse samples, the use of dynamic and ecologically relevant stimuli that simulate naturalistic interactions, and a focused examination of GenAI performance in processing separate sensory channels^50,51^. These gaps are particularly crucial in the context of mental health applications, where GenAI`s ability to accurately interpret emotional cues could transform clinical practice by enabling scalable, cost-effective tools for screening, monitoring, and supporting therapeutic processes^50–52^. The present study aims to address these gaps through a comparative research design, examining the emotion recognition capabilities of three GenAI models relative to human performance benchmarks. For this purpose, a substantial set of validated stimuli from the EU-Emotion Stimulus Set^53,70^, comprising dynamic vocal and bodily stimuli, was utilized.

The field of research into the emotion recognition capabilities of GenAI is in its nascent stages, a state of knowledge that necessitates strict adherence to rigorous scientific standards. For this reason, and with the aim of strengthening the validity and reliability of our findings, the research hypotheses and analysis plan were pre-registered. The pre-registration procedure was preceded by an initial data collection and analysis phase, which provided an empirical basis for formulating the final hypotheses. Subsequently, a new and separate dataset was collected for the main study, with its analysis commencing only after the completion of the pre-registration process. Finally, we pre-registered the following hypotheses: (H1) The overall accuracy of GenAI models will not differ significantly from human accuracy in interpreting emotions from vocal tone and body gestures; (H2) compared to human performance, GenAI models will exhibit a positivity bias, demonstrating significantly higher accuracy for positive emotions than for negative emotions; (H3) human participants will show no significant difference in recognition accuracy between positive and negative emotions; (H4) GenAI models will demonstrate a positivity bias in emotion recognition; (H5) advanced GenAI models will outperform less advanced models in emotion recognition accuracy; and (H6) GenAI models will achieve higher recognition accuracy from vocal stimuli than from bodily gesture stimuli. Hypothesis pre-registration and data are available via https://osf.io/hypbe/overview?view_only=aa6eb8e4cc24489fbfcf65428cccb5ab.

## Results

### Hypothesis 1: GenAI vs. human overall accuracy

#### Bodily gesture modality

**Fig. 1:**
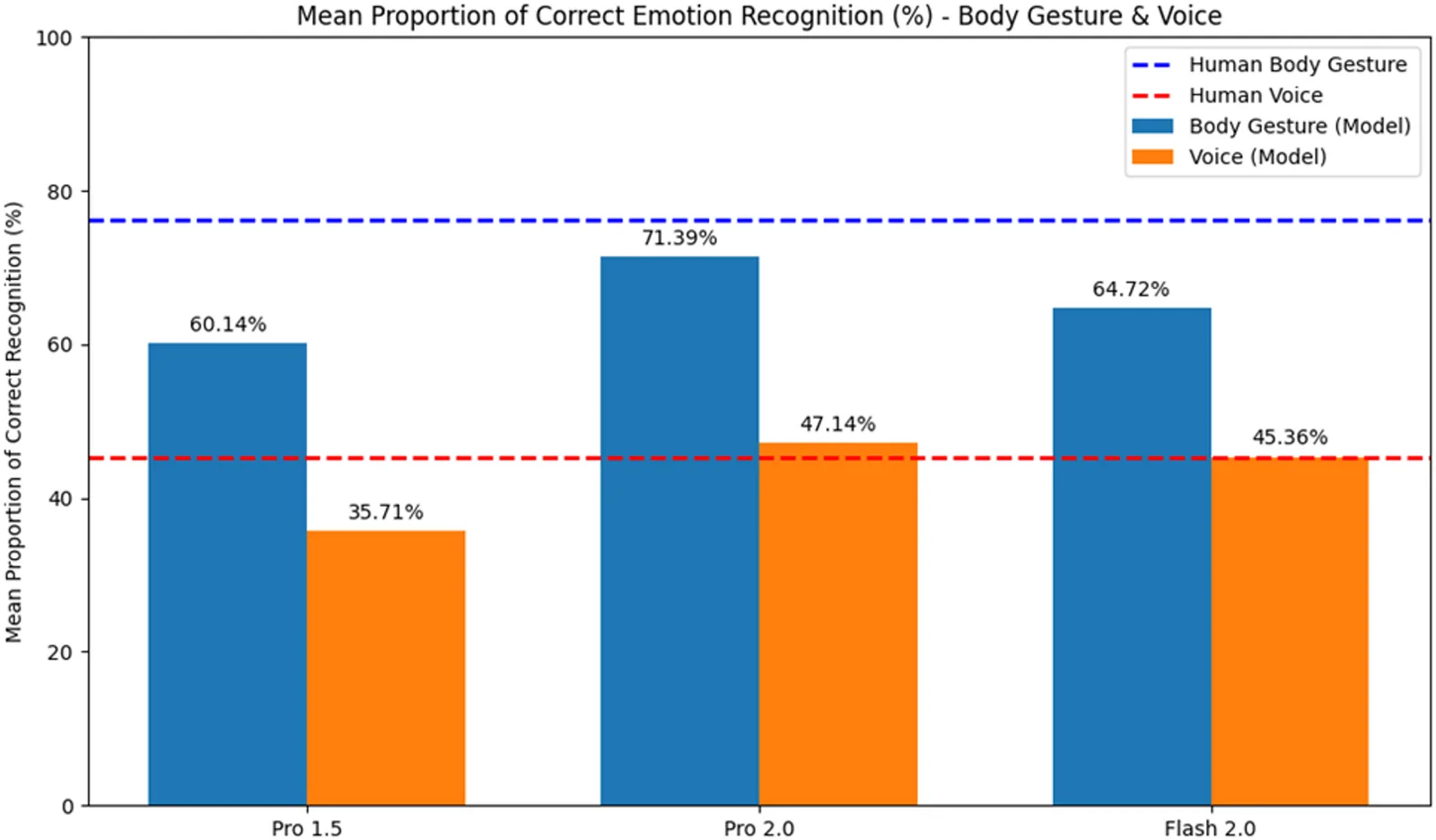
Mean proportion of correct emotion recognition (%) for bodily-gesture and vocal stimuli in human observers and three generative artificial intelligence (GenAI) models.

A two-proportion z-test was conducted to compare the recognition accuracy of AI models and human participants in the bodily gesture modality. Human participants demonstrated a correct recognition rate of 77.08%. The Flash 2 model achieved a recognition rate of 64.72%, which was significantly lower than that of humans (*z* = -6.87, *p* < .001), with a medium effect size (h = 0.51). The Pro 2 model achieved 71.39%, which was also significantly lower than human performance (*z* = -3.22, *p* = .001, *h* = 0.25, representing a small effect). The Pro 1.5 model yielded 60.14%, significantly lower than humans (*z* = -9.32, *p* < .001) with a medium-to-large effect (*h* = 0.72). Among the AI models, Pro 2 closely approximated human performance. **See** Fig. 1.

#### Vocal modality

In the vocal modality, human participants achieved a recognition rate of 45.19%. Flash 2 produced a similar recognition rate of 45.36%, with no significant difference from human performance (*z* = 0.07, *p* = .943, *h* = 0.003). The Pro 2 model achieved a slightly higher accuracy of 47.14%, which was not significantly different from human accuracy (*z* = 0.81, *p* = .416, *h* = 0.04). In contrast, Pro 1.5 demonstrated a lower recognition rate of 35.71%, significantly below human performance (*z* = -4.00, *p* < .001, *h* = 0.19, reflecting a small effect). These results indicate that Flash 2 and Pro 2 demonstrated performance comparable to humans, with no statistically significant differences observed, whereas Pro 1.5 exhibited significantly lower performance compared to humans. **See** Fig. 1.

### Hypothesis 2: GenAI vs. human accuracy by emotional valence

Emotional stimuli were categorized into valence-based groups (positive and negative) according to ratings provided in the original dataset aligning with the study’s primary objective of examining valence-specific performance biases. The neutral category (*n* = 2 for each modality) was excluded from the primary inferential analyses to maintain this dichotomous focus, and because its insufficient sample size precluded adequately powered statistical comparisons. However, given the clinical importance of detecting neutral or flat affect, an exploratory descriptive analysis of these specific stimuli is provided in the Supplementary Material. For bodily gesture stimuli, the positive group comprised 14 items, whereas the negative group included 20. For vocal stimuli, the positive group consisted of 14 items, and the negative group comprised 26.

#### Bodily gestures

**Fig. 2:**
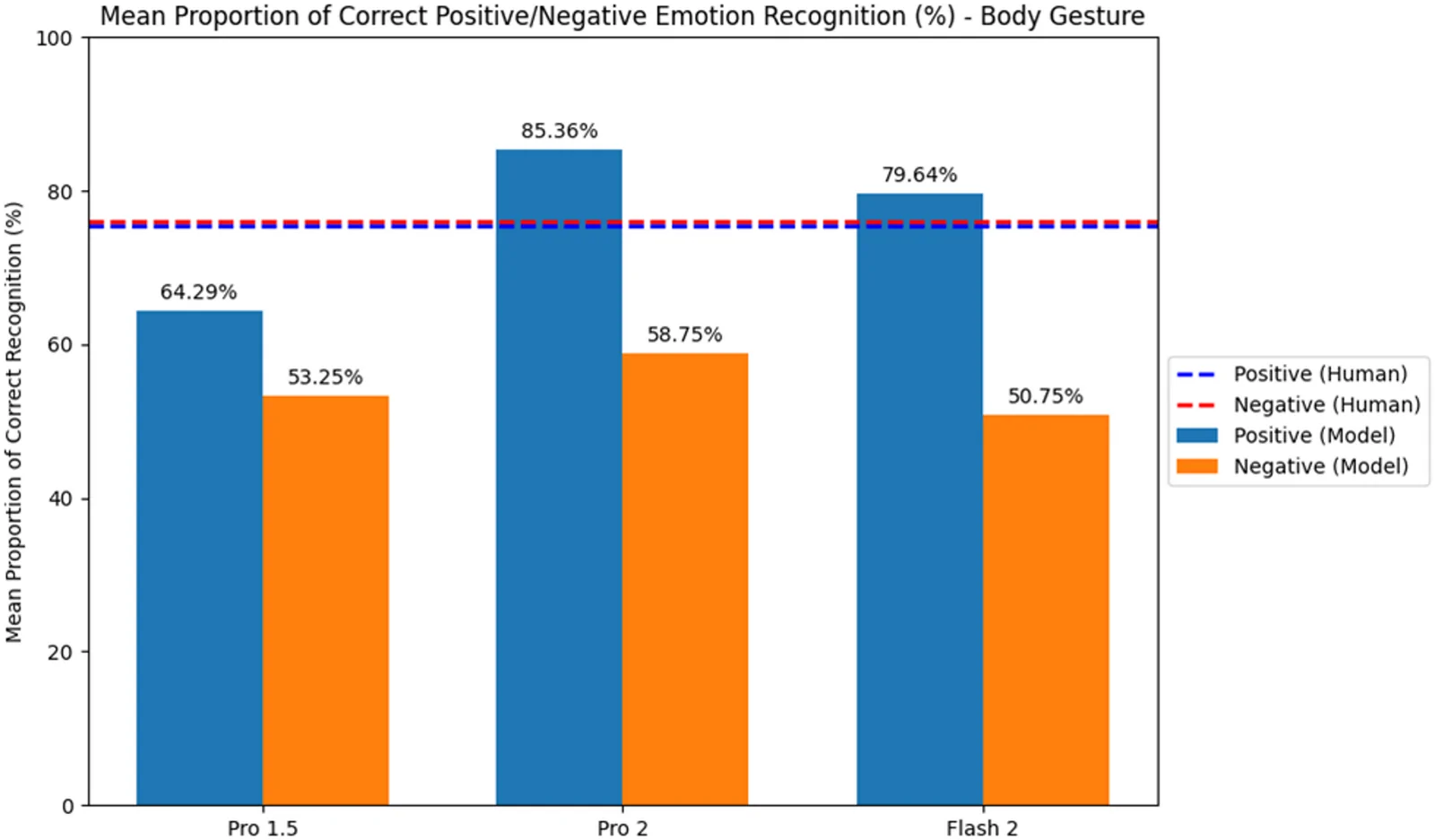
Mean proportion of correct emotion recognition (%) for positive and negative bodily-gesture stimuli in human observers and three GenAI models. Note. Blue bars depict positive-valence stimuli; orange bars depict negative-valence stimuli. Dotted lines indicate human performance for positive (blue) and negative (red) stimuli.

For positive expressions, human accuracy was 76.01%. The Pro 2 model significantly outperformed humans (85.36%, *p* < .001), representing small effect (*h* = 0.24), while Flash 2 was comparable (79.64%, *p* = .196, *h* = 0.09) and Pro 1.5 was significantly worse (64.29%, *p* < .001) with a small effect (*h* = 0.26). For negative expressions, human accuracy was 76.97%. All AI models underperformed significantly, with Flash 2 at 50.75%, Pro 2 at 58.75%, and Pro 1.5 at 53.25% (all *p* < .001, with effect sizes ranging from *h* = − 0.39 to *h* = -0.55, representing small to medium effects). **See** Fig. 2**and** Table 1.

**Table 1:** Comparison of GenAI and human emotion recognition accuracy by valence and modality.

Modality	Comparison	z-value	*p*-value	Effect Size (h)
Bodily Gestures				
	Within-Group Comparisons (Positive vs. Negative)			
	Human	-0.60	0.549	-
	Flash 2	7.67	< 0.001	0.62
	Pro 2	7.43	< 0.001	0.61
	Pro 1.5	2.87	0.004	0.22
	Between-Group Comparisons (GAI vs. Human)			
	Flash 2 vs. Human (Positive)	1.29	0.196	0.09
	Flash 2 vs. Human (Negative)	-10.47	< 0.001	-0.55
	Pro 2 vs. Human (Positive)	3.39	< 0.001	0.24
	Pro 2 vs. Human (Negative)	-7.41	< 0.001	-0.39
	Pro 1.5 vs. Human (Positive)	-4.01	< 0.001	-0.26
	Pro 1.5 vs. Human (Negative)	-9.52	< 0.001	-0.50
Vocal Expressions				
	Within-Group Comparisons (Positive vs. Negative)			
	Human	-0.22	0.824	-
	Flash 2	3.81	< 0.001	0.28
	Pro 2	2.43	0.015	0.18
	Pro 1.5	-0.77	0.444	-0.05
	Between-Group Comparisons (GAI vs. Human)			
	Flash 2 vs. Human (Positive)	2.82	0.004	0.24
	Flash 2 vs. Human (Negative)	-1.00	0.316	-0.06
	Pro 2 vs. Human (Positive)	1.88	0.058	0.16
	Pro 2 vs. Human (Negative)	-0.62	0.534	-0.04
	Pro 1.5 vs. Human (Positive)	-2.23	0.025	-0.19
	Pro 1.5 vs. Human (Negative)	-2.41	0.016	-0.15

#### Vocal expressions

**Fig. 3:**
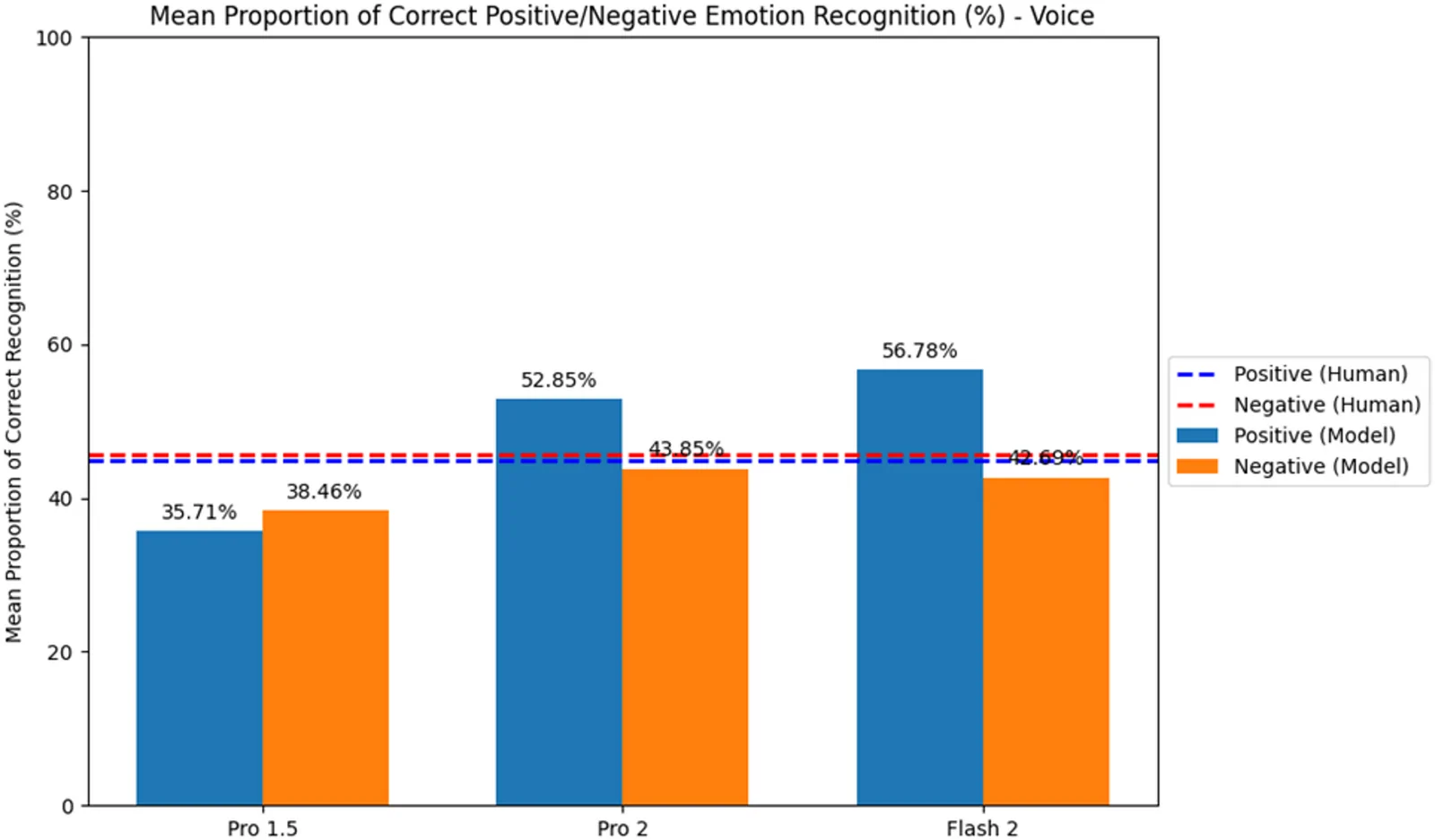
Mean proportion of correct emotion recognition (%) for positive and negative vocal stimuli in human observers and three GenAI models. Note. Blue bars represent positive-valence stimuli; orange bars represent negative-valence stimuli. Dotted lines indicate human performance for positive and negative stimuli.

For positive expressions, human accuracy was 44.91%. Flash 2 significantly outperformed humans (56.78%, *p* = .004) with small effect (*h* = 0.24). Pro 2 had higher accuracy but without significance difference (52.85%, *p* = .058), and Pro 1.5 was significantly worse (35.71%, *p* = .025). For negative expressions, human accuracy was 45.74%. Flash 2 (42.69% *p* = .316) and Pro 2 (43.85% *p* = .534) were not significantly different from humans, while Pro 1.5 was again significantly worse (38.46%, *p* = .016, *h* = -0.15), though this comparison did not retain significance following FDR correction (see Method). See Table 1; Fig. 3.

### Hypothesis 3: human accuracy by emotional valence

Two-proportion z-tests were conducted to examine differences in human recognition accuracy between positive and negative stimuli. For bodily gestures, accuracy for positive (76.01%) and negative (76.97%) stimuli was not significantly different (*z* = − 0.60, *p* = .549). Similarly, for vocal stimuli, accuracy for positive (44.91%) and negative (45.74%) expressions was not significantly different (*z* = − 0.22, *p* = .824). No significant differences were found for humans across emotional valences in both modalities.

### Hypothesis 4: GenAI accuracy by emotional valence

Two-proportion z-tests compared each AI model’s accuracy for positive versus negative stimuli.

#### Bodily gestures

All models were significantly more accurate for positive than negative gestures: Flash 2 (79.64% vs. 50.75%, *p* < .001, *h* = 0.62, a medium-to-large effect), Pro 2 (85.36% vs. 58.75%, *p* < .001, *h* = 0.61), and Pro 1.5 (64.29% vs. 53.25%, *p* = .004, *h* = 0.22, reflecting a small effect).

#### Vocal expressions

Flash 2 (56.78% vs. 42.69%, *p* < .001, *h* = 0.28) and Pro 2 (52.85% vs. 43.85%, *p* = .015, *h* = 0.18) were also significantly more accurate for positive than negative vocalizations, both representing small effect sizes. Pro 1.5 showed no significant difference (35.71% vs. 38.46%, *p* = .444, *h* = -0.05).

These findings, detailed in Table 1, reveal a consistent positivity bias in the Flash 2 and Pro 2 models.

### Hypothesis 5: overall performance differences between GenAI models

Kruskal-Wallis tests were conducted to evaluate differences in recognition performance across GenAI models. No significant differences were observed for either bodily gesture stimuli, *H*(2) = 0.75, *p* = .686, or vocal stimuli, *H*(2) = 2.61, *p* = .270.

### Hypothesis 6: GenAI accuracy by modality

**Fig. 4:**
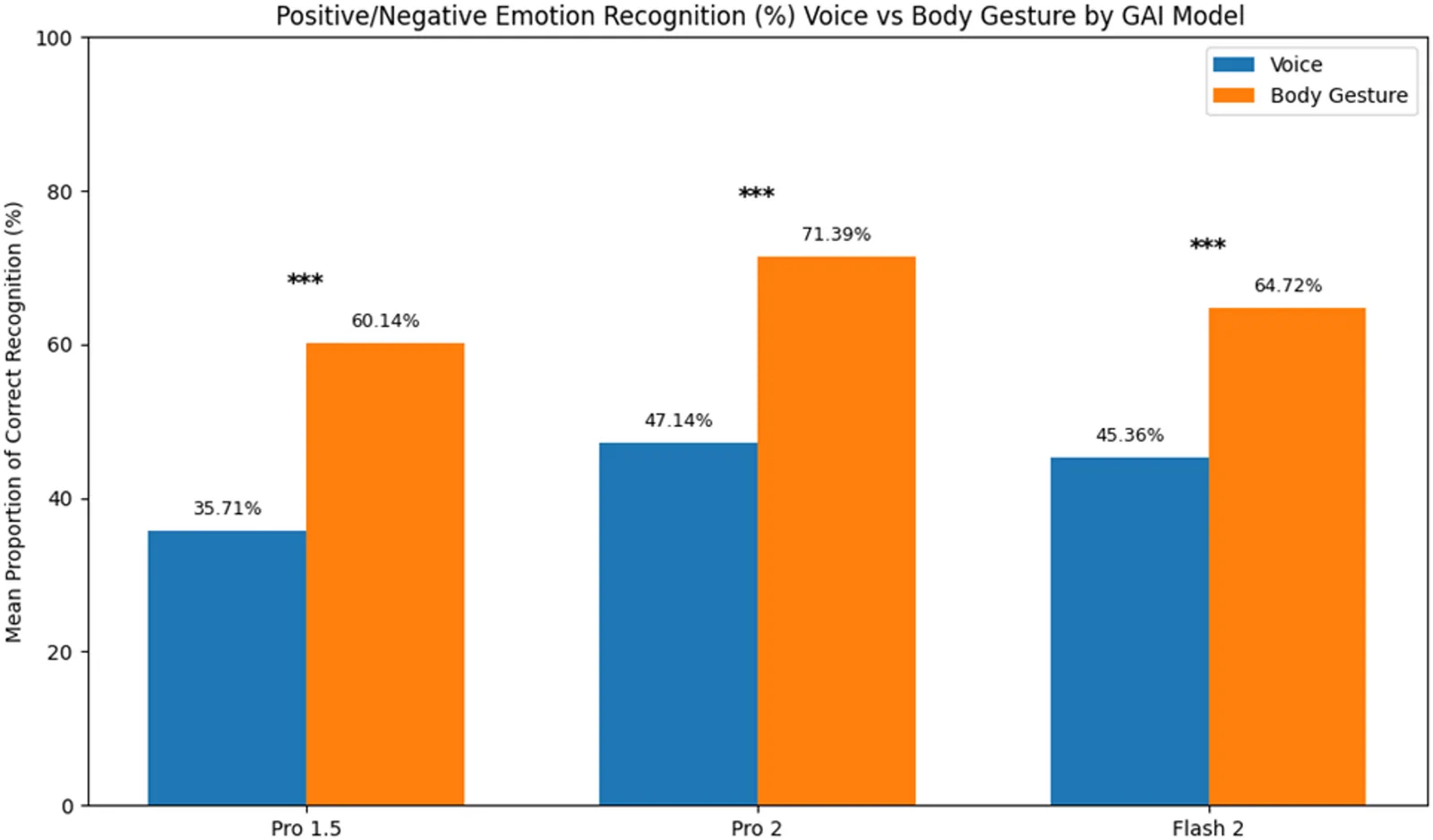
Comparison of GenAI recognition accuracy between bodily gesture and vocal expression modalities across all three models. Note: *** *p* < .001.

Two-proportion z-tests were conducted to compare the accuracy of each GenAI model across modalities. Contrary to H6, which predicted higher accuracy for vocal stimuli, the results indicated the opposite pattern. Flash 2 showed significantly higher accuracy for bodily gestures (64.72%) compared to vocal expressions (45.36%; *z* = 7.65, *p* < .001, *h* = 0.39). Similarly, Pro 2 exhibited greater recognition for bodily gestures (71.39%) than for vocal expressions (47.14%; *z* = 9.68, *p* < .001, *h* = 0.50). Pro 1.5 followed the same pattern, with significantly better performance for bodily gestures (60.14%) than for vocal stimuli (35.71%; *z* = 9.64, *p* < .001, *h* = 0.49). These findings indicate consistent and medium-to-large modality effects favoring bodily gestures across all models. (**See** Fig. 4**)**

### Confusion Matrix Analysis

To provide a more granular characterization of model performance beyond the valence-level analyses, confusion matrices were computed for all three models across both modalities (see Supplementary Material). In the bodily gesture modality, several positive emotions including Excited, Interested, Joking, and Kind achieved perfect or near-perfect recognition rates across all models, while Happy was perfectly recognized by Pro 2 and Flash 2 but was entirely misclassified as Surprised by Pro 1.5. In contrast, recognition failures were concentrated among negative emotions. Afraid was systematically confused with Ashamed (37.5%-55%), Sad with Disgusted (30%–100%), and Angry with Disgusted in Flash 2 and Pro 1.5 (45%-91.9%). Hurt was never correctly identified by any model, being classified as Surprised and Unfriendly (Pro 2 and Flash 2) or as Happy and Unfriendly (Pro 1.5).

In the vocal modality, Disappointed achieved perfect recognition across all models, while Interested and Worried were recognized with high accuracy in the advanced models but showed reduced performance in Pro 1.5 and Flash 2, respectively. Jealous and Sneaky were uniformly misclassified as Disappointed across all three models. Proud was never correctly recognized by any model, and Hurt was entirely misclassified as Unfriendly by Pro 1.5 while being only partially recognized by Pro 2 and Flash 2. Notably, vocal misclassifications clustered along prosodically similar groupings rather than distributing randomly across categories. Full confusion matrices are presented in the Supplementary Material (Figures S1–S6).

## Discussion

This study provides an empirical examination of the proficiency of contemporary GenAI models in recognizing emotions from dynamic body and vocal stimuli, benchmarked against human performance using a substantial, validated dataset. Our findings offered only partial support for our primary hypothesis (H1), which predicted that the overall accuracy of GenAI models would not differ significantly from human accuracy in interpreting emotions from both vocal tone and body gestures. The support for this hypothesis was modality-dependent: in line with H1, advanced models like Gemini Pro 2 and Flash 2 achieved near-human accuracy in deciphering vocal emotional cues. However, contradicting H1, their ability to interpret bodily gestures lagged significantly behind human benchmarks, with all models performing statistically worse than the human observers. Critically, and in alignment with our second hypothesis (H2), a discernible and statistically significant bias towards superior recognition of positive over negative emotions permeated GenAI performance. This pattern mirrors emerging evidence of positivity biases in large-language-model emotion recognition^49^. This asymmetry was particularly salient in the bodily gesture modality and was starkly contrasted with human responses, which were notably balanced and consistent, and reveals a fundamental limitation of current models, carrying direct implications for their ecological validity and safe deployment. Furthermore, all GenAI models exhibited significantly greater accuracy with bodily gesture stimuli compared to vocal expressions, in contrast to our hypothesis that GenAI models will achieve higher recognition accuracy from vocal stimuli than from bodily gesture stimuli. Collectively, these results delineate a nuanced and empirically substantiated profile of GenAI’s current social-cognitive capacities and, more importantly, expose an inherent asymmetry in their processing of affective information that carries profound implications.

The investigation into GenAI’s capacity to interpret emotions from dynamic bodily gestures ventures into relatively uncharted territory. To our knowledge, this study constitutes one of the first systematic assessments of GenAI performance utilizing such decontextualized, dynamic bodily stimuli from a validated corpus. The accurate decoding of these subtle cues is integral to adaptive social functioning^11^. The observation that models, particularly Gemini Pro 2, achieved performance metrics approaching human levels with bodily gestures (albeit with a significant overall deficit) signals considerable latent potential. Crucially, within this modality, GenAI models consistently manifested a statistically significant predilection for positive emotions over negative ones (supporting H4 for bodily gestures). This contrasted with the human data, in which no statistically significant difference in accuracy was found (supporting H3), thereby highlighting a distinct GenAI specific processing bias. This represents not merely a quantitative difference but a qualitative gap, indicating a fundamental divergence in the processing mechanisms of AI compared to human social cognition. The models underperformed humans on negative expressions yet showed less disparity, and in the case of Pro 2, even superiority, on positive ones. From a mental health perspective, this algorithmic positivity bias mandates a critical examination of its ethical and clinical implications. While GenAI’s capacity to interpret kinesic cues could prospectively inform assistive technologies for therapists or serve as feedback mechanisms in clinical training, evidence suggests that such biases can propagate to human users and amplify existing cognitive distortions^54^. A system that systematically demonstrates reduced sensitivity to nonverbal distress cues could lead to severe diagnostic failures and compromise therapeutic reliability^33^. This is particularly concerning as the “working alliance” between therapist and patient, a key predictor of therapeutic success, is built on the patient’s perception of being accurately seen and understood^55^. An AI tool that overlooks the slumped posture of despair or the fidgeting of anxiety fails at this foundational level of attunement, lacking the nuanced understanding and empathy human therapists provide^20,36^. Such algorithmic ‘blindness’ to negative affect could lead to significant clinical risks, for instance, by misinterpreting or missing crisis signals, underestimating suicide risk, providing misleading responses, hallucinating content, or prematurely terminating high-risk conversations^19,35,37,56^. This highlights a major ethical barrier; addressing such algorithmic biases and safety limitations is not merely a technical adjustment but a central prerequisite for the safe and responsible deployment of generative AI in mental health^19^.

The assessment of GenAI’s proficiency in discerning emotion from isolated vocal prosody interrogates another vital facet of nonverbal communication. Vocal tone provides the indispensable paralinguistic “music” that accompanies the linguistic script, modulating social exchanges and conveying the true emotional meaning that words alone often conceal^38,57^. It is intrinsically linked to human mentalization^11^ and is fundamental to the therapeutic alliance, where a therapist’s attunement to a patient’s subtle vocal shifts, such as flatness in depression or tremors in anxiety is a cornerstone of accurate empathy and diagnosis. Our finding that Gemini Pro 2 and Flash 2 performed comparably to humans with vocal stimuli is therefore highly significant. Yet, even in this domain of relative GenAI strength, the clinical implications are complicated by our second key finding: these models displayed a persistent and statistically significant advantage for positive vocal expressions over negative ones (supporting H4), a pattern absent in balanced human performance (supporting H3). This finding indicates that the positivity bias is not modality-dependent but is likely a systemic feature of the models themselves, rooted in either their architecture or training data. From a clinical psychology perspective, the implications of this profile are twofold. On one hand, a GenAI proficient in vocal analysis could offer unprecedented tools for clinical training and practice^8,58^. On the other hand, the positivity bias presents a serious risk. This condition exposes the danger of algorithmic dis-empathy or misattunement; an AI system that fails to detect the flat affect of depression or the strained tone of anxiety could fundamentally misunderstand a patient’s state, potentially leading to harmful clinical inaction or inappropriate automated responses^33,34^. Such a system undermines the principle of validation, a cornerstone of therapy^15^. In practice, this could manifest as an AI-powered diagnostic tool repeatedly failing to flag early-stage depression characterized by subtle vocal flattening, given AI’s documented struggles with nuanced interpretation and reliability across different groups^20,37,59^. Alternatively, an automated support bot might respond with inappropriate optimism to a user whose vocal tremors clearly signal profound distress, reflecting limitations in contextual understanding, empathy, and crisis recognition^19,20,36,60^. This creates a risk of “automation bias” or diagnostic overshadowing, where the AI’s potentially biased or inaccurate output might cause a human clinician to second-guess their own intuition^35^, thereby delaying critical intervention^19^. As with kinesic analysis, understanding and mitigating this bias is a prerequisite for any responsible application in mental healthcare. The confusion matrices for the vocal modality provide indirect but informative evidence bearing on this question. Since the vocal stimuli consisted of pseudo-sentences devoid of semantic content, any audio-to-text transcription would yield meaningless strings carrying no emotional information, and errors would be expected to distribute randomly across categories. Instead, misclassifications clustered along acoustically interpretable dimensions: Jealous and Sneaky were uniformly classified as Disappointed across all models, consistent with shared low-arousal, subdued prosodic contours; Afraid was confused with Disappointed or Unfriendly, states sharing tense, constrained vocal qualities; and Hurt was confused with Surprised or Unfriendly rather than distributing randomly. This systematicity is more consistent with prosodic feature extraction than with a text-conversion account, though it does not definitively rule out hybrid processing pathways. This remains an important direction for future mechanistic research.

The broader implications of this demonstrated positivity bias are substantial and extend beyond clinical applications. As AI systems become more integrated into socially embedded contexts, their ability to understand and respond to the full spectrum of nonverbal cues will be crucial^58^ and to prevent the recursive amplification of affective biases documented in human–AI feedback loops^54^. The current study highlights not only the “black box” challenge but also the need to investigate the origins of this bias examining both the training data and the models optimization mechanisms. Is this positivity bias a product of the over-representation of positive interactions in training corpora, an artifact of the safety and preference-based fine-tuning process, or does it reflect a deeper architectural preference for low-error solutions associated with more unambiguous, positive categories? This question is paramount. The origin of this bias is likely multifactorial, though the available evidence permits a tentative prioritization among candidate mechanisms. The most probable primary contributor is the Reinforcement Learning from Human Feedback (RLHF) fine-tuning process, which explicitly optimizes for user-perceived helpfulness and safety, systematically rewarding agreeable and non-confrontational outputs while penalizing responses that engage with negative or distressing content^49,56,62^. This is supported by the observation that the positivity bias was most pronounced in the advanced models (Pro 2 and Flash 2), which have undergone more extensive alignment tuning, whereas Pro 1.5 while less accurate overall did not exhibit a valence difference in the vocal modality. Moreover, training data imbalance represents a likely contributing factor: large-scale corpora suffer from a well-documented over-representation of positive expressions relative to subtle negative affect^61^, which would establish a baseline skew that alignment procedures subsequently amplify. An architectural explanation wherein the models inherently favor low-ambiguity, high-confidence categories that happen to be positive is less parsimonious but cannot be excluded, particularly given the consistent finding across both modalities. An AI that is systematically biased towards positivity may be perceived as more “agreeable” or “helpful” in low-stakes interactions, but in high-stakes social, educational, or therapeutic roles, this same bias can become a form of selective ‘algorithmic blindness’, failing to identify and respond precisely to the most critical signals of distress. The challenge of sourcing comprehensive, diverse, and ethically-sourced multimodal datasets is therefore not just technical, but deeply ethical, as it directly impacts the social-cognitive “attunement” of the GenAI systems we are building.

### Limitations

Several limitations warrant consideration. First, the use of enacted emotional expressions^53,63,70^ may not fully capture the subtlety of spontaneous emotions, potentially limiting ecological validity^32^. Second, findings from these three models may not generalize to other GenAI architectures or future versions. At the time of study design and pre-registration, the Gemini family uniquely offered public access to natively multimodal architectures capable of processing both dynamic video and isolated vocal tone. However, the rapid proliferation of advanced multimodal models necessitates caution in generalizing these specific accuracy rates. Third, presenting stimuli in isolation deviates from authentic, multimodal social perception. Fourth, the forced-choice response format simplifies the emotion recognition task. Fifth, the exclusion of the neutral category from the primary valence analyses initially limited conclusions about GenAI’s processing of ambiguous states; however, this was partially mitigated through our exploratory supplementary analysis, though the findings remain constrained by the limited representation. Additionally, an imbalance favoring negative stimuli was present in the stimulus set. Sixth, the use of zero-shot prompting methodology means that performance could potentially be enhanced through different configurations or fine-tuning, which were beyond this study’s scope. Finally, the human benchmark data were derived from validation studies published in 2016 and 2019, whereas the GenAI evaluation was conducted in 2025. However, human accuracy in basic emotion recognition from nonverbal cues reflects well-established perceptual capacities that have demonstrated high temporal stability^64^, and we do not anticipate that contemporaneous data collection would yield substantively different benchmarks.

### Future research directions

Future research should employ more ecologically valid stimuli, such as clips from real-world interactions or assessments like the MASC^65^. Investigations should expand to include complex social emotions and cross-cultural expressions^24^. A key priority is to elucidate the underlying mechanisms of GenAI’s processing, for instance, by disentangling lexical from prosodic cues in vocal stimuli. Longitudinal studies tracking model evolution are needed, as is research on the impact of different training strategies on performance and bias. Specifically, future investigations must test newer and more diverse models from other developers (e.g., OpenAI models, Anthropic’s Claude) to determine whether the positivity bias observed here is influenced by Gemini’s specific native multimodal architecture, or if it represents a ubiquitous artifact of industry-wide alignment processes. Critically, these potential sources can be empirically distinguished through targeted approaches. Comparing the outputs of base models prior to alignment with their fine-tuned counterparts would isolate the contribution of RLHF from pre-training data imbalances. Mechanistic interpretability techniques, including analysis of neural network activations and attention weight visualization could reveal whether models consistently assign lower attention to cues indicative of clinical severity. Evaluating emotional granularity would clarify whether the bias reflects a computational difficulty in discriminating among complex negative emotions. Systematic cross-model comparisons spanning different developers and architectures would establish whether this positivity bias is industry-wide or developer-specific.

In addition, future work should explore alternative prompting strategies such as chain-of-thought prompting, which encourages stepwise reasoning and may elicit more transparent and cognitively plausible decision-making processes in GenAI systems. Incorporating cognitive-scientific “thinking models” such as dual process frameworks or models of emotion appraisal may provide a theoretically grounded lens for designing prompts, interpreting AI outputs, and aligning generative responses with human-like reasoning. These approaches may not only enhance performance but also offer valuable insights into how GenAI processes and represents emotional information. Finally, rigorous ethical examination of these advancing capabilities is paramount to address concerns of privacy, bias, and psychological impact on users^6,66^.

## Method

### Study design

The present study employed a comparative design to assess the emotion recognition capabilities of three GenAI models within the Gemini family: Gemini Pro 1.5, Gemini Pro 2 (Experimental), and Gemini Flash 2^67^. These models were selected based on their status as the most advanced publicly accessible GenAI systems available at the time of data collection, which was conducted between February and March 2025. The decision to focus exclusively on the Gemini family was made to maintain methodological consistency, allowing for a controlled comparison across models built on a shared, natively multimodal architecture well-suited for the study’s audio-visual stimuli.

### Generative AI models

The Gemini family of models is inherently multimodal, designed from the ground up to process and reason across a combination of data types, including text, images, audio, and video. This native multimodality distinguishes them from earlier models that were primarily text-based with added visual capabilities.

Gemini 1.5 Pro is a powerful and versatile model known for its large context window, allowing it to process vast amounts of information (up to 1 million tokens). It is designed for complex reasoning tasks that require a deep understanding of the provided context^67^.

Gemini Flash 2 represents a model optimized for speed and efficiency. This model maintains a robust 1 million token context window. It is designed for high-frequency or low-latency tasks, providing rapid responses while maintaining multimodal capabilities, making it suitable for more interactive, real-time applications^67^.

Gemini Pro 2 (Experimental) was an experimental version of the next-generation Pro model available during the testing period. It offered access to more advanced reasoning and multimodal understanding capabilities that were in development, building upon the foundation of the 1.5 Pro model^67^.

### Model temperature

A series of two-proportion z-tests were conducted to examine the effects of temperature settings on AI model performance (Pro 1.5, Pro 2, Flash 2) for bodily gesture stimuli. In large language models, temperature is a parameter that controls the randomness of the model’s output: lower values (e.g., 0.5) produce more deterministic and conservative responses, whereas higher values (e.g., 1.0) allow for more diverse and exploratory outputs. No significant differences in recognition accuracy were observed across temperature levels for any of the models when applied to bodily gesture stimuli: Flash 2 (*z* = -0.17, *p* = .868), Pro 2 (*z* = -0.29, *p* = .769), and Pro 1.5 (*z* = -0.17, *p* = .871). A comparable analysis for vocal stimuli likewise revealed no significant differences in performance for Flash 2 (*z* = -0.44, *p* = .658) and Pro 1.5 (*z* = -0.36, *p* = .720). However, the Pro 2 model demonstrated a significantly higher recognition rate at a temperature setting of 0.5 (*z* = -2.75, *p* = .005). Based on this finding, a temperature of 0.5 was selected for all subsequent analyses involving vocal stimuli to both optimize performance for the temperature-sensitive model and ensure consistency across comparisons. For bodily gesture stimuli, the default temperature setting of 1.0 was retained, as no model exhibited temperature-dependent variation in performance.

### Procedure

The evaluation protocol was designed to assess the emotion recognition performance of GenAI models under standardized and replicable conditions. All model assessments were conducted using a structured prompt-based interface in the Gemini API Pro developer environment.

The three GenAI models were evaluated independently under standardized conditions. The evaluation was automated via a continuous script execution with each of the 20 trials per stimulus processed as a strictly independent, stateless API call. No conversational history or context was retained between prompts. During each session, the model received a visual or audio stimulus (either a video or audio clip), followed by a multiple-choice question that replicated the fixed-response format used in the human testing paradigm. A zero-shot prompting approach was employed, such that models were not provided with any prior examples, fine-tuning, or contextual training before the task.

### Stimulus materials

Stimulus materials were drawn from the EU-Emotion Stimulus Set and EU-Emotion Voice Database, a validated audiovisual database developed to assess multimodal emotion recognition^53,63,70^. The bodily gesture stimuli comprised dynamic video clips performed by 19 multi-ethnic professional actors (10 female, 9 male; ages 10–70) recruited from UK acting agencies and drama schools. To constrain perceptual information to kinesic cues, actors were filmed from the neck down against a neutral background, with all facial expressions and vocalizations excluded. The stimuli depict full-body portrayals of discrete emotional states, performed at a high intensity according to standardized scripts. While most stimuli featured a single actor, six inherently social emotions (e.g., ashamed, jealous) were portrayed in dyadic interactions, reflecting the social context required for their expression. The vocal stimuli were drawn from the database’s English-language collection, featuring 18 native British English-speaking actors (9 female, 9 male; ages 10–70) from UK professional acting agencies and drama schools. A key methodological feature of this set is the use of pseudo-sentences, devoid of semantic content, to isolate emotional prosody. By having actors vocalize these meaningless phrases to convey specific emotions (e.g., happiness, anger), the affective information was methodologically constrained to the paralinguistic channel (i.e., tone, pitch, and rhythm). Recordings were conducted in a soundproof studio, with intensity levels (high and low) systematically varied for basic emotions.

The present study employed the EU-Emotion Stimulus Set and EU-Emotion Voice Database^53,63,70^ to examine the capacity of various Gemini models to recognize emotional expressions conveyed through bodily gestures and vocal tone, assessed independently. To select a subset of these materials for the current study, we first identified stimuli for which prior human recognition rates ranged between 40% and 95%, a criterion intended to mitigate potential ceiling or floor effects in model performance. To ensure multiple representations for each emotion, we then selected two distinct stimuli (e.g., two different video clips for “happiness”) for every emotion that had at least two examples meeting the accuracy criterion. This number was chosen to balance the representativeness of each emotion with the overall number of emotions included, as requiring more than two stimuli per emotion would have significantly reduced the number of emotions available for testing from the original database. This selection process resulted in a final set of thirty-six video clips, representing 18 distinct emotions, and forty-two audio recordings, representing 21 distinct emotions. The difference in the total number of stimuli between modalities reflects the number of emotions in the original datasets that met our inclusion criteria.

### Human benchmark and ethical considerations

Human recognition benchmarks were derived from the original large-scale validation studies of the EU-Emotion Stimulus Set for bodily gestures and the EU-Emotion Voice Database for vocal expressions, which included samples of *N* = 1,231 and *N* = 427, respectively. The present study did not involve recruitment of new human participants, collection of new human-subject data, or any intervention involving human participants. Instead, it relied on EU-Emotion stimuli obtained upon request to the database developers and provided for scientific research purposes only, in accordance with their access conditions, which preclude redistribution of the materials. Additional inputs comprised previously published aggregate human benchmark data from the original validation studies and newly generated performance data from GenAI models. Ethical approval and informed consent procedures for the original human validation studies were handled within those studies. Because the present analyses used only previously published aggregate benchmark data and did not involve new human-subject data collection, no additional institutional ethics approval or informed consent procedure was required. All methods in the present study were carried out in accordance with relevant guidelines and regulations.

#### Participants: bodily gestures

According to the original validation study^53,70^ the data was collected from a sample of 1,231 participants (803 female, 428 male). The average age was 44 years (SD = 16.7, range: 16–84). Participants were recruited through university mailing lists, research databases, and online social media. The stimuli were divided across multiple online surveys; therefore, each individual stimulus was rated by a subset of the total participant pool, with a minimum of 54 participants evaluating each survey.

#### Participants: vocal expressions

The benchmark for vocal prosody relies on the UK-specific validation data from the EU-Emotion Voice Database^63^. This group of participants is independent of the one that validated the bodily gesture stimuli. The UK validation data was collected from a sample of 427 participants (283 females). The average age was 38 years (range: 18–90). Recruitment was conducted through similar channels, including university mailing lists and research participant databases. Because the voice stimuli were distributed across 20 different surveys in the UK, each stimulus was rated by a unique subset of the total sample, with at least 20 participants completing each survey.

### GenAI evaluation protocol

The evaluation protocol was designed to assess the emotion recognition performance of the GenAI models under standardized and replicable conditions. All model assessments were conducted via the Gemini API, using Google Colab notebooks to interact with the Google AI Studio interface. The three GenAI models were evaluated independently, with all testing conducted in a single uninterrupted session per model.

To ensure consistency and comparability with the human benchmark, each model was administered the identical six-alternative forced-choice (6-AFC) recognition task from the original validation studies. For each stimulus, the model was presented with a multiple-choice question consisting of the target emotion, four pre-selected distractor emotions, and a “none of the above” option. A zero-shot prompting approach was employed, such that models were not provided with any prior examples, fine-tuning, or contextual training before the task.

Two categories of stimuli were used: (1) video clips displaying bodily gestures and (2) audio clips containing vocal tone. For each modality, the models were evaluated across two temperature settings (0.5 and 1.0) to examine variability in output due to sampling stochasticity. Two independent trials were conducted per temperature level for each model, resulting in a total of four testing blocks per model. Each model completed 20 independent trials for each emotion stimulus in both the bodily and vocal conditions, yielding 4,320 iterations for bodily stimuli and 5,040 for vocal stimuli. For each trial, the model’s response was parsed to extract one of the six predefined emotion labels. Responses were logged and subsequently aggregated for analysis.

To assess performance, we calculated the aggregate recognition accuracy for both human and GenAI groups using a pooled frequency approach. In order to account for the varying number of human raters per stimulus in the original validation studies and prevent potential weighting biases, we summed the total number of correct identifications (*x*) against the total number of observations (*n*) within each modality. This method ensures that the two-proportion z-tests accurately reflect the cumulative evidence across the dataset, inherently weighting the contribution of each stimulus by its specific sample size. For the bodily gesture modality, the human benchmark comprised *n* = 2511 total observations, while the GenAI models were evaluated across *n* = 720 trials per model. For the vocal modality, the human benchmark included *n* = 1102 observations, with GenAI models completing *n* = 840 trials per model. This approach maintains strict comparability and ensures that the statistical comparisons are grounded in the actual distribution of responses.

### Assessment of training data contamination

It should be noted upfront that the EU-Emotion Stimulus Set^53,70^ and the EU-Emotion Voice Database^63^ are not freely publicly available and are accessible for scientific use subject to the access conditions specified by the database developers, making it highly unlikely that the models were trained on the specific stimulus materials used in the present study. To empirically evaluate whether the GenAI models had prior exposure to the EU-Emotion stimulus materials used in this study, we conducted a series of diagnostic prompts prior to the emotion recognition tasks. Without presenting the actual stimuli, we queried each model’s ability to (a) identify the EU-Emotion Stimulus Set or EU-Emotion Voice Database based on a structured description of the materials, (b) list items or actor portrayals from established emotion recognition datasets, and (c) correctly label brief, stylized descriptions of prototypical stimuli. Across all three models, none demonstrated specific knowledge of the EU-Emotion stimuli. However, some models did correctly identify the RAVDESS (Ryerson Audio-Visual Database of Emotional Speech and Song)^68^ when queried, suggesting possible exposure to similar but distinct emotional datasets. Importantly, none of the models reproduced the specific items, labels, or actor identities associated with the EU-Emotion materials. Moreover, as detailed in the Results section, model performance remained imperfect and included systematic errors, indicating that task performance was unlikely to reflect direct training contamination or memorized content.

### Statistical data analysis plan

All analyses were conducted using Python version 3.11 with appropriate libraries for statistical computing and visualizations (Pandas, matplotlib). The analysis plan detailed below follows the pre-registered protocol. Given violations of normality in recognition accuracy distributions (as indicated by Shapiro–Wilk tests), non-parametric and distribution-free methods were employed. Specifically, two-proportion z-tests were used to assess differences in recognition accuracy between groups, and Kruskal–Wallis tests were employed for multi-group comparisons across AI models. Any deviations from the initial statistical plan were made after a more in-depth examination of the data structure, which revealed complexities not evident in the preliminary review. The statistical tests for each hypothesis are detailed in Table 2. Effect sizes for differences in proportions were evaluated using Cohen’s *h*, with values of 0.20, 0.50, and 0.80 interpreted as small, medium, and large effects, respectively (Cohen, 1988)^69^. All hypotheses and statistical tests were pre-registered prior to data collection. Nevertheless, a Benjamini–Hochberg FDR correction was applied across all comparisons as a robustness check. All results retained significance, with one exception: the difference between Pro 1.5 and human accuracy for positive vocal expressions did not survive correction. This does not affect the study’s primary conclusions. Uncorrected results are reported in the main text, consistent with the pre-registered analysis plan.

**Table 2:** Summary of statistical tests and hypotheses.

Statistical Test Employed	Purpose / Comparison	Relevant Hypothesis(es)	Independent Variable(s)	Dependent Variable	Effect Size Metric
Two-proportion z-test	Compare GAI model accuracy to human performance overall and by emotional valence (positive vs. negative)	H1, H2	Group (GAI model vs. Human), Emotional Valence (for H2)	Recognition Accuracy Rate	Cohen’s h
Two-proportion z-test	Compare human accuracy across emotional valence conditions (positive vs. negative)	H3	Emotional Valence (Positive/Negative)	Recognition Accuracy Rate	Cohen’s h
Two-proportion z-test	Compare accuracy within each GAI model across emotional valence conditions (positive vs. negative)	H4	Emotional Valence (Positive/Negative)	Recognition Accuracy Rate	Cohen’s h
Kruskal–Wallis test	Examine overall recognition performance differences across the GAI models (Flash 2, Pro 2, Pro 1.5) for each modality	H5	GAI Model	Recognition Accuracy Rate	N/A
Two-proportion z-test	Compare the accuracy of each GAI model across modalities (bodily gestures vs. vocal expressions)	H6	Modality (Bodily Gestures vs. Vocal Expressions)	Recognition Accuracy Rate	Cohen’s h

### Ethics and funding declarations

The ethical status of the human benchmark data is described in the Methods section. The current study did not receive any funding.

## Conclusion

This study provides an important empirical screening of current GenAI capabilities in recognizing emotions from body language and vocal tone. While demonstrating promising advancements, with some models approaching human-level performance in specific contexts, the findings also reveal persistent challenges, including performance gaps relative to humans and a notable positivity bias. Specifically, in the bodily gesture modality, human participants outperformed all GenAI models overall, although the most advanced model (Gemini Pro 2) approached human-level accuracy. In contrast, for vocal stimuli, both Gemini Pro 2 and Flash 2 matched but did not significantly exceed human accuracy. However, these results must be interpreted with caution, as they were not consistently statistically significant.

Crucially, all GenAI models demonstrated a systematic positivity bias, showing significantly higher accuracy for positive emotional expressions compared to negative ones in both modalities, a pattern not observed in human participants. This bias was particularly pronounced in bodily gesture recognition, where AI models significantly underperformed relative to humans for negative emotions but occasionally exceeded human performance for positive ones. Such asymmetries underscore critical differences in how artificial systems versus human observers process affective cues. Future research employing diverse, ecologically valid stimuli and transparent methodologies is essential to fully understand the boundaries, validity, and potential societal impact of GenAI ‘s burgeoning social-cognitive skills.

## Supplementary Information

Below is the link to the electronic supplementary material.

Supplementary Material 1

## Data Availability

The dataset generated and analyzed during the current study are available in the OSF repository via https://osf.io/hypbe/overview? view_only=aa6eb8e4cc24489fbfcf65428cccb5ab. The original EU-Emotion stimulus files are not publicly shared, in accordance with the access conditions of the database developers.
