# Supplementary material for: Generative AI emotion recognition from bodily gestures and vocal tone reveals modality-specific performance and positivity bias
Source: Sci Rep. 2026 Sep 3;16:27663. doi: 10.1038/s41598-026-58013-z (PMC13542163; doi:10.1038/s41598-026-58013-z)
Supplement: Supplementary file 1 [file 41598_2026_58013_MOESM1_ESM.docx]

**Supplementary Materials**

| **Section** | **Description** | **Page** |
| --- | --- | --- |
| **Supplementary Text** |  |  |
| **SI 1** | Analysis of Neutral Stimuli | 2 |
| **SI 2** | Confusion Matrices for Emotion Recognition | 2 |
| **Supplementary Figures** |  |  |
| **Figure S1** | Body gestures modality: Confusion matrix for Gemini Pro 2 | 3 |
| **Figure S2** | Body gestures modality: Confusion matrix for Flash 2 | 4 |
| **Figure S3** | Body gestures modality: Confusion matrix for Gemini Pro 1.5 | 5 |
| **Figure S4** | Vocal modality: Confusion matrix for Gemini Pro 2 | 6 |
| **Figure S5** | Vocal modality: Confusion matrix for Flash 2 | 7 |
| **Figure S6** | Vocal modality: Confusion matrix for Gemini Pro 1.5 | 8 |

**Supplementary Information 1. Analysis of Neutral Stimuli**

Primary analyses in the main manuscript focused exclusively on comparing positive and negative emotional valences to investigate potential positivity biases, given the clinical importance of detecting neutral or flat affect (particularly in conditions such as depression), an exploratory analysis of the neutral stimuli (*n* = 2 per modality) was conducted. Although the severely restricted sample size fundamentally limits statistical power and necessitates cautious interpretation, exploratory inferential comparisons with False Discovery Rate (FDR) corrections were performed to provide preliminary insights.

For bodily gestures, human recognition accuracy for neutral stimuli was *M* = 83.06% (*SD* = 0.58%). In comparison, all GenAI models achieved perfect recognition rates: Flash 2 (*M* = 100.00%, *SD* = 0.00%), Pro 2 (*M* = 100.00%, *SD* = 0.00%), and Pro 1.5 (*M* = 100.00%, *SD* = 0.00%). This recognition rate was significantly higher than human performance across all three models (*p* = .047).

Conversely, for tone expressions, human accuracy was *M* = 40.45% (*SD* = 0.64%). The GenAI models struggled significantly with this modality. Both Flash 2 and Pro 1.5 failed to recognize neutral vocal tone (*M* = 0.00%, *SD* = 0.00%), performing significantly worse than human benchmarks (*p* = .032 for both models). Pro 2 demonstrated higher accuracy (*M* = 50.00%, *SD* = 70.71%), with no statistically significant difference from human performance (*p* = .880).

**Supplementary Information 2:** **Confusion Matrices for Emotion Recognition**

The following confusion matrices present the detailed emotion-by-emotion recognition patterns for each GenAI model across both modalities. Figures S1-S3 display the bodily gesture modality and Figures S4-S6 display the vocal modality. In each matrix, rows represent the true (intended) emotion and columns represent the model’s predicted emotion. Cell values indicate the percentage of trials in which a given true emotion was classified as each predicted category.

**Bodily Gesture Modality**

***Figure S1.*** *Confusion matrix for Gemini Pro 2 - Bodily Gesture modality. Rows represent true emotions; columns represent predicted emotions. Values indicate the percentage of trials classified into each category.*

***Figure S2.*** *Confusion matrix for Gemini Flash 2 - Bodily Gesture modality. Rows represent true emotions; columns represent predicted emotions. Values indicate the percentage of trials classified into each category.*

***Figure S3.*** *Confusion matrix for Gemini Pro 1.5 - Bodily Gesture modality. Rows represent true emotions; columns represent predicted emotions. Values indicate the percentage of trials classified into each category.*

**Vocal Modality**

***Figure S4.*** *Confusion matrix for Gemini Pro 2 - Vocal modality. Rows represent true emotions; columns represent predicted emotions. Values indicate the percentage of trials classified into each category.*

***Figure S5.*** *Confusion matrix for Gemini Flash 2 - Vocal modality. Rows represent true emotions; columns represent predicted emotions. Values indicate the percentage of trials classified into each category.*

***Figure S6.*** *Confusion matrix for Gemini Pro 1.5 - Vocal modality. Rows represent true emotions; columns represent predicted emotions. Values indicate the percentage of trials classified into each* *category.*
